# Poor-quality antimalarials further health inequities in Uganda

**DOI:** 10.1093/heapol/czz012

**Published:** 2019-12-09

**Authors:** Daniel R Evans, Colleen R Higgins, Sarah K Laing, Phyllis Awor, Sachiko Ozawa

**Affiliations:** 1 Duke University School of Medicine, DUMC 3710 Durham, NC 27710, USA; 2 Division of Practice Advancement and Clinical Education, UNC Eshelman School of Pharmacy, University of North Carolina at Chapel Hill, CB#7574, Beard Hall 115H, Chapel Hill, NC 27599, USA; 3 Department of Community Health and Behavioural Sciences, Makerere University School of Public Health, Mulago Hospital Complex, Mulago Hill, P.O. Box 7072, Kampala, Uganda; 4 Department of Maternal and Child Health, UNC Gillings School of Global Public Health, University of North Carolina, 135 Dauer Dr., Chapel Hill, NC 27599, USA

**Keywords:** Malaria, health inequities, antimalarial, quality, substandard, falsified, agent-based model, Uganda

## Abstract

Substandard and falsified medications are a major threat to public health, directly increasing the risk of treatment failure, antimicrobial resistance, morbidity, mortality and health expenditures. While antimalarial medicines are one of the most common to be of poor quality in low- and middle-income countries, their distributional impact has not been examined. This study assessed the health equity impact of substandard and falsified antimalarials among children under five in Uganda. Using a probabilistic agent-based model of paediatric malaria infection (Substandard and Falsified Antimalarial Research Impact, SAFARI model), we examine the present day distribution of the burden of poor-quality antimalarials by socio-economic status and urban/rural settings, and simulate supply chain, policy and patient education interventions. Patients incur US$26.1 million (7.8%) of the estimated total annual economic burden of substandard and falsified antimalarials, including $2.3 million (9.1%) in direct costs and $23.8 million (7.7%) in productivity losses due to early death. Poor-quality antimalarials annually cost $2.9 million to the government. The burden of the health and economic impact of malaria and poor-quality antimalarials predominantly rests on the poor (concentration index −0.28) and rural populations (98%). The number of deaths among the poorest wealth quintile due to substandard and falsified antimalarials was 12.7 times that of the wealthiest quintile, and the poor paid 12.1 times as much per person in out-of-pocket payments. Rural populations experienced 97.9% of the deaths due to poor-quality antimalarials, and paid 10.7 times as much annually in out-of-pocket expenses compared with urban populations. Our simulations demonstrated that interventions to improve medicine quality could have the greatest impact at reducing inequities, and improving adherence to antimalarials could have the largest economic impact. Substandard and falsified antimalarials have a significant health and economic impact, with greater burden of deaths, disability and costs on poor and rural populations, contributing to health inequities in Uganda.

**Key Messages**

- Substandard and falsified antimalarials results in $26 million in patient costs to Ugandan children each year.
- The health and economic burden resulting from poor-quality antimalarials rests primarily on poor and rural populations.
- The poorest wealth quintile paid 12.1 times that of the wealthiest quintile for substandard and falsified antimalarials.
- Interventions to improve antimalarial quality had the greatest impact on reducing inequities.

## Background

Health equity is defined as everyone having ‘the opportunity to attain their full health potential’ and no one being ‘disadvantaged from achieving this potential because of their social position or other socially determined circumstance’ (Brennan Ramirez *et al*., 2008). Health inequities arise when a socially determined group or groups of a population are prevented from achieving their full health potential. For example, when individuals in lower socio-economic statuses (SESs) face higher risks for poor health outcomes, it creates an inequitable division in health that is attributable to a predetermined social circumstance. Health inequities have a significant health and socio-economic impact, creating and reinforcing cycles of poverty and poor health, and slowing national development (WHO, 2018a). Preventing and addressing health inequities is important because all individuals should have the opportunity to attain their highest level of health (WHO, 2018a).

Malaria poses a significant threat to achieving health equity. The malaria burden disproportionately affects low- and middle-income countries (LMICs), and rests on children, the poor and rural populations (Filmer, 2005; Roca‐Feltrer *et al*., 2008; Uganda Bureau of Statistics (UBOS) and ICF, 2018). The World Health Organization (WHO) estimates that globally, 219 million malaria cases and 435 000 deaths were due to malaria in 2017 (WHO, 2018c). The brunt of this burden rests on countries in Sub-Saharan Africa where 90% of these cases and 91% of these deaths occur (WHO—AFRO, 2017; WHO, 2017c). Uganda ranks third in Sub-Saharan Africa in total number of malaria cases (Roberts and Matthews, 2016). Children under five are the most vulnerable to malaria infection and death, representing two-thirds (285 000) of malaria deaths (Maitland, 2016; WHO, 2018b). As a result, malaria is one of the leading causes of under-five mortality in Uganda (Roberts and Matthews, 2016; UNICEF, 2018). The 2016 Uganda Demographics and Health Survey (DHS) found that the prevalence of malaria among children in the lowest wealth quintile were over 10 times higher than the prevalence of children in the highest quintile [Uganda Bureau of Statistics (UBOS) and ICF, 2018]. Similarly, children in rural households were three times more likely to be infected with malaria than urban children [Uganda Bureau of Statistics (UBOS) and ICF, 2018].

Substandard and falsified medicines also disproportionately affect LMICs due to poor pharmaceutical governance and supply chain management in these settings (Newton *et al*., 2010; Nayyar *et al.*, 2012; WHO, 2017b). As defined by the WHO, substandard medicines are ‘authorized medical products that fail to meet either quality standards, specifications, or both’; and falsified medicines are ‘medical products that deliberately or fraudulently misrepresent their identity, composition or source’ (WHO, 2017a). Antimalarials are one of the medication classes most commonly found to be of poor quality (Newton *et al*., 2010; WHO, 2017b). On the basis of a recent meta-analysis, 19.1% of antimalarials in LMICs were identified to be substandard or falsified (Ozawa *et al.*, 2018), and prevalence as high as 35% have been reported in Uganda (Bate *et al*., 2008; WHO, 2009, 2017b). These medications contribute to the malaria burden by prolonging illness and increasing the patient’s risk of treatment failure, progression to severe malaria, and death (Newton *et al.*, 2006; Amin and Kokwaro, 2007; Newton *et al.*, 2011; Nayyar *et al.*, 2012; Buckley and Gostin, 2013; WHO, 2017b). By reducing clinical efficacy, substandard and falsified antimalarials also increase the risk of antimicrobial resistance and add to healthcare expenditures (Buckley and Gostin, 2013; Chaccour *et al*., 2015; WHO, 2017b).

Through a model that examines the heterogeneity of a paediatric population at a country level, this study assesses the inequitable divisions in health outcomes as well as direct and indirect economic costs that result from substandard and falsified antimalarials. We estimate the burden of disease, death and disability, along with the costs to patients and society of substandard and falsified antimalarials. We present results by SES and urban/rural regions to examine the burden specific to poor and rural populations. Through scenario analysis, we also examine the benefits of potential interventions to reduce the burden of health inequities for paediatric malaria in Uganda.

## Materials and methods

We developed and utilized the SAFARI (Substandard and Falsified Antimalarial Research Impact) model, a dynamic agent-based model built in NetLogo (Version 6.0.2, Wilensky, 1999). The model is probabilistic, with an ability to simulate the natural heterogeneity that exists across population characteristics, risk of malaria infection, patient care-seeking rates, disease outcomes and associated costs. Key epidemiological, demographic, care seeking and cost data were derived from available literature and are presented in Table 1. The SAFARI model is explained in further detail in other publications (Ozawa *et al.*, 2019a; Ozawa *et al.*, 2019b), with inputs and details specific to Uganda presented here.

**Table 1.** Model inputs

Model inputs	Input	Range	Source
Demographic and epidemiological data			
<5 Population at risk	7 881 620		UN/DESA Population Division (2017)
Malaria incidence for children <5	0.447	(0.197–0.744)	Malaria Atlas Project
Asymptomatic malaria case rate	0.156	(0.08–0.23)	Roh *et al.* (2016)
Rural proportion of population	0.824		Uganda Bureau of Statistics (UBOS) and ICF (2018)
Untreated case progression to severe	0.130	(0.07–0.3)	Lubell *et al.* (2011b)
Treatment failure progression to severe	0.020	(0.005–0.05)	Das *et al.* (2013); Lubell *et al.* (2014)
Case fatality rate for a severe case receiving quinine	0.109	(0.06–0.22)	Dondorp *et al.* (2010)
Case fatality rate for a severe case receiving other treatments	0.109	(0.06–0.22)	Assumption
Case fatality rate for a severe case receiving ACTs	0.085	(0.06–0.22)	Dondorp *et al.* (2010)
Probability that a severe case pro gresses to NS	0.032	(0.028–0.035)	Dondorp *et al.* (2010)
Healthcare-seeking behaviour			
Care-seeking behaviour (%)			
Public facilities	34.7%		Uganda Bureau of Statistics (UBOS) and ICF (2015)
Private facilities	40.8%		
Pharmacies	1.0%		
Drug stores	5.6%		
CHWs	0.7%		
Self/neighbours	12.7%		
No treatment	4.3%		
Medication effectiveness			
ACTs cure rate	0.9755	(0.9615–0.9895)	Staedke *et al.* (2004); Piola *et al.* (2005, 2010); Yeka *et al.* (2005, 2008, 2013); Bukirwa *et al.* (2006); Achan *et al.* (2009); Arinaitwe *et al.* (2009); Bassat *et al.* (2009); Clark *et al.* (2010); Four Artemisinin-Based Combinations (4ABC) Study Group (2011); Verret *et al.* (2011)
Quinine cure rate	0.8818	(0.8484–0.9152)	Verret *et al.* (2011); Yeka *et al.* (2013)
Other treatment cure rate^a^	0.7167	(0.6581–0.7753)	Checchi *et al.* (2004); Bakyaita *et al.* (2005); Yeka *et al.* (2005)
No treatment cure rate	0		Assumption based on Ashley and White (2014)
Proportions of SF medications			
ACTs		Coefficient	
Not SF (API > 85%)	80.5%	1	Bate *et al.* (2008); WHO (2009); Bjorkman Nyqvist *et al.* (2012); Kitutu (2015)
Category 1: API = 75–85%	10.5%	0.75	Adjusted ACT Consortium Drug Quality Project Team and the IMPACT2 Study Team (2015); Kaur *et al.* (2015)
Category 2: API = 50–75%	4.5%	0.5	
Category 3: API <50%	4.5%	0	
Quinine			
Not SF (API > 85%)	77.9%	1	Ogwal-Okeng *et al.* (2003); Bate *et al.* (2008, 2010); WHO (2009); Bjorkman Nyqvist *et al.* (2012); Kitutu (2015)
Category 1: API = 75–85%	11.9%	0.75	Adjusted ACT Consortium Drug Quality Project Team and the IMPACT2 Study Team (2015); Kaur *et al.* (2015)
Category 2: API = 50–75%	5.1%	0.5	
Category 3: API <50%	5.1%	0	
Alternative treatments			
Not SF (API > 85%)	68.7%	1	Ogwal-Okeng *et al.* (2003); WHO (2009); Bate *et al.* (2010)
Category 1: API = 75–85%	16.9%	0.75	Adjusted ACT Consortium Drug Quality Project Team and the IMPACT2 Study Team (2015); Kaur *et al.* (2015)
Category 2: API = 50–75%	7.3%	0.5	
Category 3: API <50%	7.2%	0	
Medication stock by facility			
Public facilities			
% Stock ACTs	89.5%		Uganda Bureau of Statistics (UBOS) and ICF (2015)
% Stock quinine	9.2%		
% Stock other treatments	1.3%		
Private facilities			
% Stock ACTs	77.2%		
% Stock quinine	14.3%		
% Stock other treatments	8.6%		
Pharmacy			
% Stock ACTs	76.0%		
% Stock quinine	0.0%		
% Stock other treatments	24.0%		
Drug stores			
% Stock ACTs	80.9%		
% Stock quinine	19.1%		
% Stock other treatments	0.0%		
CHWs			
% Stock ACTs	78.9%		
% Stock quinine	0.0%		
% Stock other treatments	21.1%		
Self/neighbours			
% Stock ACTs	87.2%		
% Stock quinine	9.7%		
% Stock other treatments	3.1%		
Probability facility has antimalarial in stock			
Public facilities	96.1%		ACTwatch Group and PACE (2015)
Private facilities	88.6%		
Pharmacies	99.7%		
Drug stores	86.1%		
CHWs	99.7%		
Self/Neighbour	100.0%		Assumption
Costs^b^			
Patient out-of-pocket costs			
Public facilities/CHWs			Assumption based on Uganda Bureau of Statistics (UBOS) and ICF (2015)
Average cost of ACTs	$0.00		
Average cost of quinine	$0.00		
Average cost of other treatments	$0.00		
Private facilities			
Average cost of ACTs	$2.59	(1.48–3.99)	ACTwatch Group and PACE (2015)
Average cost of quinine	$3.39	(2.75–4.08)	
Average cost of other treatments	$0.65	(0.49–0.82)	ACTwatch Group and PACE (2015)
Pharmacies			
Average cost of ACTs	$2.91	(1.55–4.69)	
Average cost of quinine	$2.72	(2.10–3.42)	
Average cost of other treatments	$0.48	(0.32–0.66)	ACTwatch Group and PACE (2015) Assumption based on Uganda Bureau of Statistics (UBOS) and ICF (2015) Assumption
Drug stores			
Average cost of ACTs	$1.62	(1.05–2.31)	
Average cost of quinine	$3.39	(2.76–4.08)	
Average cost of other treatments	$0.48	(0.33–0.66)	
Self/Neighbour			
Average cost of ACTs	$0.00		
Average cost of quinine	$0.00		Assumption
Average cost of other treatments	$0.00		Assumption Batwala *et al.* (2011)Orem *et al.* (2012)
Transport (public, private)	$0.47	(0.39–0.55)	
Transport (pharmacies, drug stores)	$0.08	(0.04–0.12)	
Special foods for child	$1.15	(0.87–1.43)	Hansen *et al.* (2017)
Supplemental medicines	$1.14	(1.02–1.26)	Batwala *et al.* (2011)
Average testing costs	$0.91	(0.65–1.17)	ACTwatch Group and PACE (2015)
Private facility consultation costs	$4.35	(0–21.00)	Uganda Bureau of Statistics (UBOS) and ICF (2009)
Cost per paediatric malaria hospitalization	$14.17	(0.75–47.50)	Uganda Bureau of Statistics (UBOS) and ICF (2009)
Productivity losses			
Opportunity cost of time (public, private)	$1.73	(1.66–1.80)	Batwala *et al.* (2011)
Opportunity cost of time (pharmacies, drug stores)	$0.43	(0.01–0.80)	Orem *et al.* (2012)
Productivity losses per sick day	$1.59	(0.4–3.70)	The World Bank (2018)
Productivity losses from death	$14 959.66		The World Bank (2018)
NS disability productivity losses	$6189.87		The World Bank (2018); Vos *et al.* (2016)

ACT, artemisinin combination-based therapy; API, active pharmaceutical ingredient; CHW, community health worker; NS, neurological sequelae; SF, substandard and falsified.

^a^Other treatment included Sulfadoxine-pyrimethamine (SP), Chloroquine (CQ) and Amodiaquine (AQ).  ^b^All costs are presented in US$2017.

The model simulated 1000 children over a 1-year time horizon by 5-day increments. Each child was assigned six demographic characteristics (age, sex, location, province, SES and maternal education level) based on population distributions derived as proportions from 2014-15 Uganda Malaria Indicator Survey (MIS) [Uganda Bureau of Statistics (UBOS) and ICF, 2015]. These characteristics were chosen as significant predictors of incidence, care-seeking, disease progression and treatment outcomes, but also to facilitate analysis by demographic groups (Ozawa *et al.*, 2019b). These demographic characteristics were used to estimate individual incidence and care-seeking probabilities for each child. Every 5 days, the children faced an individual incidence of becoming infected with malaria and becoming symptomatic. Children who became symptomatic or remained symptomatic from the previous period simulated care seeking and disease progression based on their demographic characteristics. Treatment was sought from one of six locations (public facility, private facility, pharmacy, drug store, community health worker or at home/from neighbour) or not at all. Location-based care-seeking rates were derived from MIS data [Uganda Bureau of Statistics (UBOS) and ICF, 2015]. At health facilities, patients with severe malaria were treated as inpatients, while those with uncomplicated malaria were treated as outpatients. Children with uncomplicated malaria received one of three categories of antimalarials [artemisinin combination therapy (ACT), quinine or all other alternative treatments] or faced a stock out. National rates of stock outs for each location of care were extracted from Uganda ACTwatch data (ACTwatch Group and PACE, 2015).

Simulated health outcomes varied by severity and whether care was sought. Severe malaria cases could result in death, neurological sequelae or recovery, based on rates from the AQUAMAT clinical trial (Dondorp *et al*., 2010; Lubell *et al*., 2011; Das *et al.*, 2013; Lubell *et al.*, 2014). Health outcomes of children who received antimalarials were determined by the clinical efficacy of the type of antimalarial received (Checchi *et al.*, 2004; Staedke *et al.*, 2004; Bakyaita *et al.*, 2005; Piola *et al.*, 2005; Yeka *et al.*, 2005; Bukirwa *et al.*, 2006; Yeka *et al.*, 2008; Achan *et al*., 2009; Arinaitwe *et al*., 2009; Bassat *et al*., 2009; Clark *et al*., 2010; Piola *et al*., 2010; Verret *et al*., 2011; Yeka *et al*., 2013), as well as drug quality and probability of treatment adherence (Bruxvoort *et al*., 2015). Prevalence of substandard and falsified medicines was estimated for each category of antimalarial based on the WWARN (Worldwide Antimalarial Resistance Network) database and a systematic literature search specific to Uganda (Bate *et al*., 2008; WHO, 2009; Bate *et al*., 2010; Bjorkman Nyqvist *et al*., 2012; Kitutu, 2015). Substandard and falsified antimalarials were assigned an active pharmaceutical ingredient (API) concentration where medication effectiveness decreased by API concentration (ACT Consortium Drug Quality Project Team and the IMPACT2 Study Team, 2015; Kaur *et al*., 2015). Patients who received a very poor-quality antimalarial or who had very poor adherence experienced complete treatment failure and faced a higher likelihood of progressing to severe malaria. All other patients who did not recover (i.e. did not have adequate clinical and parasitological response to treatment) experienced late clinical or parasitological failure. Since poor-quality antimalarials would not affect individuals who do not seek care, we focus on outcomes for those who sought care.

Costs were incurred throughout the simulation and estimated using the cost-of-illness approach (Hodgson and Meiners, 1982). Five categories of direct costs (medication, testing, consultation, transport and hospitalization) were incurred by patients out-of-pocket based on unit costs from the literature [Uganda Bureau of Statistics (UBOS) and ICF, 2009; Batwala *et al.*, 2011; Orem *et al.*, 2012; ACTwatch Group and PACE, 2015; Management Sciences for Health, 2016; Hansen *et al*., 2017]. Four forms of indirect costs were estimated: lost wages by caregivers, opportunity cost of time, productivity losses due to neurological sequelae and productivity losses due to premature death (Vos *et al.*, 2016; The World Bank, 2018). Productivity losses were estimated using the human capital approach (Drummond *et al.*, 2015), based on Uganda’s gross domestic product, duration of lost productivity and disability-adjusted life year (DALY) weights. All costs are presented in 2017 USD. Further explanation for calculating economic outputs can be found in other publications (Ozawa *et al.*, 2019a; Ozawa *et al.*, 2019b).

We examined the distribution of health and economic burden of malaria in addition to the distributional impact of substandard and falsified antimalarials in Uganda. Number of hospitalizations, deaths and undiscounted DALYs were estimated as well as direct and indirect costs. The impact of substandard and falsified antimalarials was analysed by comparing the baseline estimates to a scenario where treatment efficacy was not reduced due to substandard and falsified antimalarials. The distributional impact of potential artemisinin resistance was also analysed in which the clinical efficacy of ACTs was reduced to be the same level as that of other treatments. A chi-squared test was used to examine the statistical significance of health and economic disparities assessing the burden of substandard and falsified medicines and artemisinin resistance. Additionally, a concentration index was used to quantitatively assess socio-economic inequity across wealth quintiles (O’Donnell *et al*., 2007). Stata 14 was used to conduct statistical analyses (StataCorp, 2015).

To account for the variation in model inputs, we recorded available ranges of modelled parameters from the literature and probabilistically ranged them altogether. Epidemiological inputs were taken from normal distributions whereas cost inputs were parameterized from gamma distributions. The overall results provide the baseline estimate as well as the 95% confidence intervals based on sensitivity analyses. Uncertainty ranges were calculated from baseline standard deviations or variance across scenarios from 10 000 model simulations.

Finally, six interventions were simulated that impact the supply chain, antimalarial treatment policies or caregiver education to examine their impact in reducing health inequities. These scenarios included: (1) Having no antimalarial stock outs at public facilities; (2) Enacting policies so that only ACTs are offered to treat malaria; (3) Enacting policies to ensure that all ACTs in Uganda are of good quality; (4) Enacting policies to ensure that all antimalarials in Uganda are of good quality; (5) Educating patients to take ACTs over all other antimalarials; and (6) Educating patients to improve treatment adherence.

## Results

We estimated that there are annually ∼3.3 million cases of malaria in children under five in Uganda who seek treatment, resulting in 12 893 (95% CI 12 668–13 117) deaths. The resulting economic impact amounts to $333 million on patients and $32 million on the government (2017 USD). Table 2 presents the distribution of the health and economic burden of malaria in Ugandan children under five from a patient perspective, broken down by the MIS distribution of SES quintile and rural/urban location. The health burden of malaria in DALYs was significantly unevenly distributed across SES quintiles (*P* < 0.01) and concentrated in the lower SES quintiles (concentration index −0.30). The lowest SES quintile carried the largest proportion of the epidemiological impact, containing approximately a third of malaria cases (35.3%), deaths (28.6%) and DALYs (33.6%). Conversely, the highest SES quintile experienced less than 2% of the malaria cases and DALYs (1.6% and 1.8%, respectively), and only 2.2% of deaths. The health burden was also significantly concentrated in rural (*P* < 0.01) populations, with 82% of the population living in rural areas but comprising over 98% of the cases, deaths, and DALYs. Similar trends are observed in relation to the economic burden, with lower SES and rural populations experiencing a majority of the direct (concentration index −0.29) and indirect costs (concentration index −0.31). The lowest SES quintile accounted for 32.5% of out-of-pocket expenses while the highest SES quintile only accounted for 2.6%. Populations in rural areas accounted for 97.9% of the total out-of-pocket expenditures.

**Table 2.** Distribution of the health and economic burden of malaria among children under five seeking treatment in Uganda

	Health impact					Economic impact on patient/caregiver^b^		
	Children	Cases^a^	Hospitalizations	Deaths	DALYs	Direct	Indirect	Total economic impact
Total (95% CI)^c^	7 881 620	3 528 304 (3 527 862– 3 528 747)	176 744 (175 095– 178 393)	12 893 (12 668– 13 117)	1 091 211 (1 061 479– 1 120 942)	$25 119 000 (25 081 489– 25 155 626)	$308 020 000 (300 306 447– 315 733 907)	$333 139 000 (325 387 936– 340 889 532)
								
SES1	20.0%	35.3% (35.2–35.4)	33.8% (33.4–34.2)	28.6% (28.1–29.1)	33.6% (32.8–34.3)	32.5% (32.4–32.6)	33.9% (33.2–34.6)	33.8% (33.1–34.4)
SES2	20.0%	26.1% (26.07–26.18)	26.2% (25.9–26.5)	26.0% (25.6–26.5)	26.0% (25.4–26.7)	27.0% (26.9–27.03)	26.2% (25.5–26.8)	26.2% (25.6–26.8)
SES3	20.0%	24.1% (24.05–24.16)	24.9% (24.6–25.2)	27.8% (27.3–28.3)	25.2% (24.6–25.9)	22.8% (22.7–22.8)	24.9% (24.3–25.5)	24.8% (24.2–25.3)
SES4	20.0%	12.8% (12.78–12.86)	13.4% (13.2–13.5)	15.4% (15.0–15.8)	13.4% (12.9–13.8)	15.2% (15.1–15.3)	13.2% (12.8–13.6)	13.4% (13.0–13.8)
SES5	20.0%	1.6% (1.64–1.66)	1.8% (1.7–1.9)	2.2% (2.0–2.3)	1.8% (1.7–2.0)	2.6% (2.57–2.62)	1.8% (1.7–2.0)	1.9% (1.7–2.0)
Concentration Index^d^	0	−0.32	−0.31	−0.25	−0.30	−0.29	−0.31	−0.31
*P-*value^e^	+++	<0.001	<0.001	<0.001	<0.001	<0.001	<0.001	<0.001
								
Rural	82.4%	98.4% (98.3–98.4)	98.3% (97.3–99.2)	98.1% (96.9–99.2)	98.2% (96.8–99.7)	97.9% (97.8–98.1)	98.2% (96.9–99.6)	98.2% (97.0–99.4)
Urban	17.6%	1.6% (1.63–1.66)	1.7% (1.68–1.79)	1.9% (1.8–2.1)	1.8% (1.6–1.9)	2.1% (2.0–2.1)	1.8% (1.6–1.9)	1.8% (1.6–1.9)
*P*-value^e^	+++	<0.001	<0.001	<0.001	<0.001	<0.001	<0.001	<0.001

CI, confidence interval; DALYs, disability-adjusted life years; SES, socio-economic status.

^a^Cases represent the annual burden of malaria including those who sought treatment and those who did not seek treatment.

^b^Estimated economic impact on patients and caregivers due to malaria among children under five seeking treatment. This does not include the economic costs incurred by the government. All costs are presented in 2017 USD.

^c^95% confidence intervals were derived from 10,000 model runs.

^d^A negative value for the concentration index indicates that a disproportionate concentration of the impact lies in low SES populations.

^e^Compares the SES quintiles and rural/urban proportions for each variable to the proportion of the model population in each category (delineated as +++).

Substandard and falsified antimalarials were annually responsible for 1121 (8.7%) of these deaths, and 26.1 million USD (7.8%) of the economic impact of malaria through treatment costs, transportation costs, productive time lost of caregivers and years of life lost due to early death. Substandard and falsified antimalarials contributed to 2.9 million USD (8.8%) in direct costs to the Ugandan government. The distribution of the health and economic impact of substandard and falsified antimalarials is presented in Table 3. The health impact of poor-quality antimalarials in terms of DALYs lost was unequally distributed (*P* < 0.01), and had the largest impact on lower SES quintiles (concentration index −0.26) and rural populations (98%). In comparison to the highest SES quintile, the lowest SES quintile is predicted to experience 23 times as many hospitalizations and 13 times as many deaths due to poor-quality antimalarials each year. Rural populations in Uganda are also expected to experience >98% of the hospitalizations and deaths due to substandard and falsified medicines, while making up 82% of the population. We simulated 60 times as many hospitalizations and 47 times as many deaths in rural compared with urban areas. The distribution was similar for economic impact of substandard and falsified antimalarials with a substantial burden on the poor (concentration index −0.28) and rural populations (98.7%). The per person out-of-pocket expenses due to poor-quality antimalarials for the lowest SES quintile was 12.2 times as high as the highest SES quintile. Rural populations paid 10.8 times that of urban populations in per person in out-of-pocket expenses due to poor-quality antimalarials.

**Table 3.** Distribution of the health and economic impact of substandard and falsified antimalarials and potential artemisinin resistance on children under five with malaria in Uganda

	Health impact			Economic impact on patient/caregiver^a^		
	Hospitalizations	Deaths	DALYs	Direct	Indirect	Total economic impact
Baseline (95% CI)^b^	176 744 (175 095– 178 393)	12 893 (12 668– 13 117)	1 091 211 (1 061 479– 1 120 942)	$25 119 000 (25 081 489– 25 155 626)	$308 020 000 (300 306 447– 315 733 907)	$333 139 000 (325 387 936– 340 889 532)
SF drugs impact						
Total impact of SF drugs	13 919 (13 896– 13 942)	1121 (1117– 1124)	78 565 (78 228– 78 914)	$2 294 000 (2 293 273– 2 294 280)	$23 839 000 (23 765 051– 23 913 015)	$26 133 000 (26 058 325– 26 207 294)
** ** SES1	4231 **(30.4%)**	280 **(25%)**	21 596 **(27.5%)**	754 000 **(32.9%)**	6 886 000 **(28.9%)**	7 641 000 **(29.2%)**
	(4222–4240)	(279–281)	(21 479–21 713)	(753 957–754 532)	(6 856 317–69 16 516)	(7 610 275–7 671 048)
** ** SES2	3767 **(27.1%)**	291 **(26%)**	25 395 **(25.7%)**	610 000 **(26.6%)**	6 362 000 **(26.7%)**	6 972 000 **(26.7%)**
	(3760–3775)	(290–292)	(19 434–19 637)	(609 559–610 117)	(6 335 566–63 88 191)	(6 945 125–6 998 308)
** ** SES3	3856 **(27.7%)**	383 **(34.2%)**	19 533 **(19.8%)**	514 000 **(22.4%)**	7 280 000 **(30.5%)**	7 793 000 **(29.8%)**
	(3849–3864)	(3849–3864)	(26 095–26 291)	(513 569–514 064)	(7 254 024–7 304 998)	(7 767 593–7 819 062)
** ** SES4	1787 **(12.8%)**	145 **(12.9%)**	10 145 **(12.9%)**	354 000 **(15.4%)**	292 000 **(12.2%)**	3 269 000 **(12.5%)**
	(1782–1791)	(1782–1791)	(10 075–10 216)	(353 519–353 948)	(2 897 192–2 933 822)	(3 250 711–3 287 770)
** ** SES5	277 **(2%)**	22 **(1.9%)**	1690 **(1.7%)**	62 000 **(2.7%)**	396 000 **(1.7%)**	458 000 **(1.8%)**
	(276–279)	(276279)	(1070–1121)	(62 056–62 231)	(389 050–402 391)	(451 106–464 622)
** ** Concentration Index^c^	−0.284	−0.236	−0.257	−0.286	−0.278	−0.278
** ** Rural	13 690 **(98.4%)**	1098 **(98%)**	77 002 **(98%)**	2 249 000 **(98.1%)**	23 301 000 **(97.7%)**	25 550 000 **(97.8%)**
	(13 668–13 713)	(1095–1100)	(76 780–77 225)	(2 248 594–2 249 586)	(23 244 936–23 357 758)	(25 493 530–25 607 343)
** ** Urban	229 **(1.6%)**	23 **(2.1%)**	1563 **(2%)**	45 000 **(1.9%)**	538 000 **(2.3%)**	582 000 **(2.2%)**
	(227–230)	(23–23)	(1538–1588)	(44 611–44 763)	(531 224–544 148)	(575 835–588 911)
AMR impact						
Total AMR impact	10 418	884	63 035	$7 526 000	$31 190 000	$38 717 000
SES1	3724 **(35.7%)**	418 **(47.4%)**	27 246 **(43.2%)**	2 424 000 **(32.2%)**	12 509 000 **(40.1%)**	14 933 000 **(38.6%)**
	(3715–3733)	(417–419)	(27 127–27 366)	(2 423 301–2 423 986)	(12 478 111–12 539 862)	(14 901 412–14 963 848)
SES2	2591 **(24.9%)**	113 **(12.8%)**	11 621 **(18.4%)**	2 059 000 **(27.4%)**	6 376 000 **(20.4%)**	8 435 000 **(21.8%)**
	(2583–2598)	(113–114)	(11 520–11 722)	(2 058 827–2 059 487)	(6 349 813–6 402 102)	(8 408 640–8 461 589)
SES3	2344 **(22.5%)**	160 **(18.1%)**	10 928 **(17.3%)**	1 742 000 **(23.1%)**	6 657 000 **(21.3%)**	8 399 000 **(21.7%)**
	(2337–2351)	(159–161)	(10 829–11 027)	(1 741 793–1 742 374)	(6 631 097–6 682 743)	(8 372 890–8 425 117)
SES4	1549 **(14.9%)**	157 **(17.8%)**	10 465 **(16.6%)**	1 123 000 **(14.9%)**	4 803 000 **(15.4%)**	5 925 000 **(15.3%)**
	(1544–1553)	(156–158)	(10 393–10 536)	(1 122 496–1 122 999)	(4 783 920–4 783 920)	(5 906 416–5 944 406)
SES5	210 **(2%)**	35 **(3.9%)**	2774 **(4.4%)**	179 000 **(2.4%)**	846 000 **(2.7%)**	1 025 000 **(2.6%)**
	(209–212)	(35–35)	(2748–2801)	(178 480–178 687)	(839 052–852 840)	(1 017 533–1 031 527)
Concentration Index^c^	−0.310	−0.328	−0.318	−0.288	−0.319	−0.313
Rural	10 351 **(99.4%)**	872 **(98.7%)**	62 051 **(98.4%)**	7 370 000 **(97.9%)**	30 675 000 **(98.3%)**	38 045 000 **(98.3%)**
	(10 329–10 373)	(870–874)	(61 826–62 277)	(7 369 251–7 370 495)	(30 617 561–30 731 831)	(37 986 812–38 102 326)
Urban	67 **(0.6%)**	12 **(1.3%)**	983 **(1.6%)**	156 000 **(2.1%)**	516 000 (1.7%)	672 000 **(1.7%)**
	(66–68)	(11.6–12)	(958–1008)	(156 254–156 431)	(509 210–522 344)	(665 465–678 775)

Bold indicates the percentage of SF drug impact or AMR impact by socio-economic status quintile or rural/urban category.

AMR, antimicrobial resistance; CI, confidence interval, DALYs, disability-adjusted life years; SES, socio-economic status; SF, substandard or falsified.

^a^Estimated economic impact on patients and caregivers due to malaria among children under five seeking treatment. This does not include the economic costs incurred by the government. All costs are presented in 2017 USD.

^b^95% confidence intervals were derived from 10,000 model runs.

^c^A negative value for the concentration index indicates that a disproportionate concentration of the impact lies in low SES populations.

**Figure 1.**
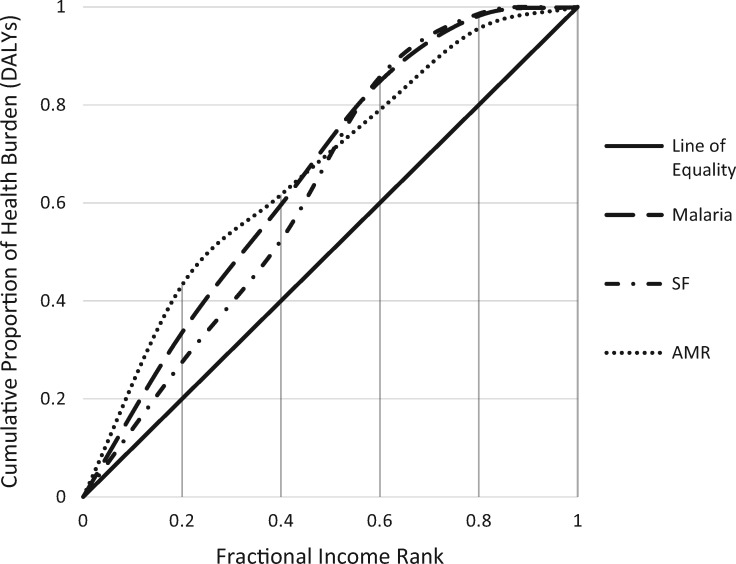
Concentration curves: distribution of the health burden of malaria, impact of substandard and falsified antimalarials, and burden of artemisinin resistance across socio-economic status quintiles. AMR, antimicrobial resistance; DALYs, disability-adjusted life years; SF, substandard and falsified.

In the event that artemisinin resistance emerges in Uganda, we estimated that the number of deaths and total economic impact on patients could increase by 6.9% and 11.6%, respectively. The potential health and economic impact of artemisinin resistance was unequally distributed (*P* < 0.01) with the majority of the potential health impact in terms of DALYs still falling on low SES (concentration index −0.32) and rural populations (98.4%). The overall economic impact was similarly placed on the poor (concentration index −0.31) and rural populations (98.3%). Compared with the burden of malaria and substandard and falsified antimalarials, the burden of artemisinin resistance was shown to result in an even greater concentration on poorer groups (Figure 1).

Among our simulated interventions, improving the quality of all antimalarials had the greatest impact, reducing the number of deaths by 8.7% (*P* < 0.01) and total economic impact by 7.8% (*P* < 0.01). Improving the quality of ACTs alone would address 71.3% of the economic impact of all poor-quality antimalarials. Ensuring that public health facilities never face a stock out had a large health and economic impact, reducing the number of deaths by 4.9% and total economic impact by $9.9 million USD. Improving treatment adherence also significantly reduced the health impact by 1425 (11%) deaths and had the largest economic impact of $30.7 million USD (9.5%). Educating caregivers to select ACTs over other treatments could potentially reduce the economic impact by $16.1 million USD (4.8%), while having health facilities only offer ACTs for malaria treatment reduced the economic impact by $17 million USD (5.1%).

**Figure 2.**
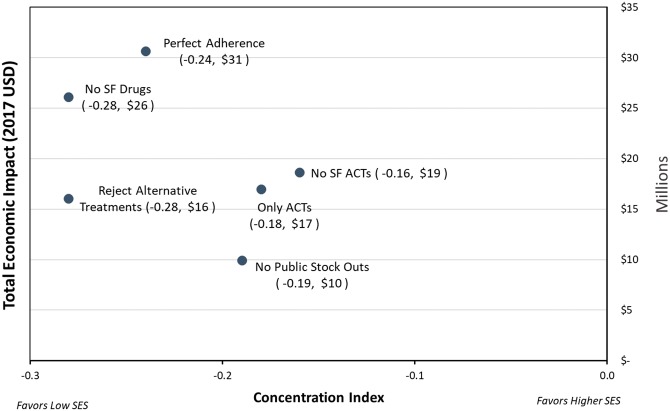
Economic impact of the modelled interventions and distribution of the impact across socio-economic status quintiles. ACTs, artemisinin combination-based therapy; SES, socio-economic status; SF, substandard and falsified

Figure 2 compares the potential economic impact of the six supply chain, policy and caregiver education interventions across SES quintiles. Each modelled intervention is expected to have an impact on equity by benefiting lower SES quintiles more (concentration indices: −0.18 to −0.28), but vary in the amount of overall economic effect in terms of direct costs to caregivers and lifetime productivity costs. The greatest overall economic impact ($30.7 million USD, 9.5%) would come from ensuring patients adhered perfectly to all anti-malarial regimens, which would require increasing the proportion of under-five perfect adherence by 25%. Improving antimalarial quality (concentration index = −0.28) and educating caregivers to reject non-ACT treatments (concentration index = −0.28) resulted in the greatest health equity effect benefitting low SES patients.

## Discussion

This study demonstrated for the first time that poor-quality antimalarials disproportionately affect low SES and rural populations. Populations in LMICs are at the greatest risk from substandard and falsified medicines due to poor supply chain management, regulations and surveillance (Newton *et al*., 2010; Nayyar *et al.*, 2012; WHO, 2017b). Further, populations in lower SESs are more likely to seek care from the informal sector, and as a result are more likely to obtain poor-quality medicines (Ogunlesi and Olanrewaju, 2010; Almuzaini *et al.*, 2013). This along with the disproportionate burden of malaria in poor populations resulted in these populations consuming a majority of poor-quality antimalarials in our simulation. This finding demonstrated that poor and rural children are at greater risk from the effects of poor-quality antimalarials. This highlights the need for pharmacovigilance and efforts to improve medicine quality by strengthening the medicine distribution chain, using quality-assured drugs, improving pharmaceutical governance and strengthening the technical capacity of regulatory laboratories, especially in poor and rural communities (Buckley and Gostin, 2013; WHO, 2017b).

Moreover, our results indicate that improving the quality of antimalarials in Uganda could also work to address health inequities. Among the scenarios we examined, removing substandard and falsified medicines and educating caregivers to reject non-ACT treatments had the largest effect on health equity, benefitting the poor and rural populations the most. Treatment adherence was shown to be important to reducing the economic burden of malaria. Even without improving medicine quality, perfect adherence of under-five patients to antimalarials could reduce the economic burden of malaria on average 9.2% across all SES levels. Improving the availability of antimalarials at public facilities resulted in a reduction in deaths (4.9%) and economic impact (3%) of malaria. To reduce stock outs and achieve improved distribution of medicines, the WHO recommends decentralizing the supply chain and implementing rigorous monitoring and evaluation systems throughout (Mabirirzi *et al*., 2014). These efforts increase access to antimalarials and reduce stock outs by identifying stock problems early, reducing not just the impact of malaria but also addressing health disparities (Buckley and Gostin, 2013; Mabirirzi *et al*., 2014). Further, efforts to improve supply chain management, and monitoring and evaluation systems could help address substandard and falsified antimalarials (Buckley and Gostin, 2013). Poor stock availability increases medication costs and creates a demand for medications in informal sectors, which in turn opens the door for substandard and falsified medicines (Buckley and Gostin, 2013). Strong monitoring and evaluation systems are necessary to prevent poor-quality medicines from penetrating the market, targeting those most at need, driving up healthcare costs, and exacerbating health disparities (Buckley and Gostin, 2013).

The findings of this study are consistent with previously reported associations between rates of malaria infection and SES or location (Roca‐Feltrer *et al.*, 2008; Nahum *et al.*, 2010; Gahutu *et al*., 2011; Roberts and Matthews, 2016). Our distribution of the malaria burden is reflective of the distribution found by the 2016 DHS and 2014-15 MIS (Uganda Bureau of Statistics (UBOS) and ICF, 2018). The WHO estimates that 3.8–8.9% of all malaria deaths result from poor-quality antimalarials in Sub-Saharan Africa, and children under five account for a high proportion (45%) of all malaria deaths (WHO, 2017b). Our estimate for under-five malaria deaths in Uganda due to substandard and falsified antimalarials (8.7%) falls in the upper end of this range.

The burden of paediatric malaria in Uganda disproportionately rests on low SES and rural populations. The reasons for the association between malaria and SES are broadly described as attributable to differences in housing structure, nutrition and education, and malaria prevention methods (Nahum *et al.*, 2010; Gahutu *et al*., 2011; Roberts and Matthews, 2016). Other studies have found that people of low SES not only have substantially higher malaria prevalence but also have greater risk of infection and catastrophic healthcare expenditures (Castillo‐Riquelme *et al*., 2008). As a result, malaria disproportionately affects the poor globally, with the poorest 20% of the world’s population accounting for a majority of malaria cases (Breman *et al*., 2004). Similar trends are observed between rural and urban populations, with children in urban areas having significantly lower risk of malaria infection [Onwujekwe *et al*., 2009; Uganda Bureau of Statistics (UBOS) and ICF, 2018]. This distribution may be explained by urban populations having higher income, education and access to healthcare and preventative measures (Onwujekwe *et al*., 2009).

Our model findings on the impact of interventions are specific to Uganda. Applications of the SAFARI model found other interventions to be more efficacious at reducing the burden of substandard and falsified antimalarials in other countries (Ozawa *et al.*, 2019a). This highlights the importance of tailoring interventions to the specific needs of a population. As such, caution should be used in extrapolating the recommended interventions to other countries.

Uganda adopted the WHO recommendation of using ACTs as the first line treatment for malaria in 2004 (WHO, 2016). As of 2016, ACTs were used to treat almost 90% of under-five malaria cases [Uganda Bureau of Statistics (UBOS) and ICF, 2018]. This success in ensuring children receive ACTs also means that poor-quality ACTs, as opposed to other antimalarials, are accounting for most of the total health and economic impact of substandard and falsified antimalarials in Uganda. Our model suggests that 71.3% of the economic impact from substandard and falsified medicines is due to low quality ACTs. Ensuring that all ACTs offered in Uganda are of high quality would significantly reduce the total burden of malaria and bring about a health equity benefit.

All statistical models are limited by the quality of their inputs and their ability to account for naturally occurring heterogeneity (Xie, 2011; Gomez-Ramirez and Sanz, 2013). We developed and utilized a probabilistic model where model parameters were derived from the most recent and highest quality published data. Further, since substandard and falsified antimalarials only affect individuals seeking treatment, this analysis primarily reports the malaria burden among cases that sought medical care. The model is limited by the lack of available data on the prevalence of substandard or falsified antimalarials. As a result the prevalence of poor-quality antimalarials for each category was not varied by care sector. We did vary incidence and care-seeking by SES and other variables. Additional data on the prevalence of substandard or falsified antimalarials by different population groups would improve our understanding of the health equity impact. Our economic impact focused on costs directly incurred by patients and caregivers to examine the distributional burden, and did not include costs incurred by government facilities. Our analysis focused only on the potential impact on the burden of malaria and did not account for the cost of implementing the interventions.

## Conclusion

We demonstrate that the burden of malaria and effects of poor-quality antimalarials disproportionately rest on poor and rural populations in Uganda. This unequal distribution exacerbates health inequities and places these populations further behind in attaining their health potential. These findings are important to inform the government, policy makers, donors and the malaria community of the issue and the impact of potential interventions to address them. Our findings indicate that efforts should focus on improving supply chain management and monitoring and evaluation systems. These efforts could improve access to medicines by increasing stock availability and reducing the prevalence of substandard and falsified antimalarials. Improving access to medicines and addressing substandard and falsified antimalarials are essential to address health inequities and reduce the health and economic burden of malaria.
